# Acetylation of XPF by TIP60 facilitates XPF-ERCC1 complex assembly and activation

**DOI:** 10.1038/s41467-020-14564-x

**Published:** 2020-02-07

**Authors:** Jiajia Wang, Hanqing He, Binbin Chen, Guixing Jiang, Liping Cao, Haiping Jiang, Guofei Zhang, Jianxiang Chen, Jun Huang, Bing Yang, Chun Zhou, Ting Liu

**Affiliations:** 1 Department of Cell Biology, and Department of General Surgery of Sir Run Run Shaw Hospital, Zhejiang University School of Medicine, Hangzhou, 310058 China; 2 The MOE Key Laboratory of Biosystems Homeostasis & Protection and Innovation Center for Cell Signaling Network, Life Sciences Institute, Zhejiang University, Hangzhou, 310058 Zhejiang China; 3 Department of General Surgery, Sir Run Run Shaw Hospital, Zhejiang University School of Medicine, Hangzhou, 310058 Zhejiang China; 4 Department of Medical Oncology, The First Affiliated Hospital, Zhejiang University School of Medicine, Hangzhou, 310058 Zhejiang China; 5 Department of Thoracic Surgery, The Second Affiliated Hospital, Zhejiang University School of Medicine, Hangzhou, 310058 Zhejiang China; 6 Department of Radiotherapy, Institute of Cancer Research and Basic Medical Sciences of Chinese Academy of Sciences, Cancer Hospital of University of Chinese Academy of Sciences, Zhejiang Cancer Hospital, Hangzhou, 310022 Zhejiang China

**Keywords:** Cancer, Cell biology

## Abstract

The XPF-ERCC1 heterodimer is a structure-specific endonuclease that is essential for nucleotide excision repair (NER) and interstrand crosslink (ICL) repair in mammalian cells. However, whether and how XPF binding to ERCC1 is regulated has not yet been established. Here, we show that TIP60, also known as KAT5, a haplo-insufficient tumor suppressor, directly acetylates XPF at Lys911 following UV irradiation or treatment with mitomycin C and that this acetylation is required for XPF-ERCC1 complex assembly and subsequent activation. Mechanistically, acetylation of XPF at Lys911 disrupts the Glu907-Lys911 salt bridge, thereby leading to exposure of a previously unidentified second binding site for ERCC1. Accordingly, loss of XPF acetylation impairs the damage-induced XPF-ERCC1 interaction, resulting in defects in both NER and ICL repair. Our results not only reveal a mechanism that regulates XPF-ERCC1 complex assembly and activation, but also provide important insight into the role of TIP60 in the maintenance of genome stability.

## Abstract (web summary)

The XPF-ERCC1 heterodimer is an endonuclease involved in nucleotide excision (NER) and interstrand crosslink (ICL) repair in mammalian cells. Here, the authors provide insights into its regulation by revealing that TIP60 regulates XPF-ERCC1 complex assembly and activation.

## Introduction

The integrity of genomic DNA is continuously assaulted by endogenous and exogenous DNA-damaging agents that cause various types of DNA lesions^1,2^. These DNA lesions can directly compromise vital cellular processes such as transcription, translation, and replication^1,2^. To cope with the detrimental effects of DNA damage, living organisms have evolved a range of highly conserved surveillance and repair mechanisms, such as nucleotide excision repair (NER), interstrand crosslink (ICL) repair, and DNA double-strand break (DSB) repair^1,2^. Defects in the regulation of any of these mechanisms often lead to genome instability, a hallmark of cancer and aging^1,2^.

The XPF-ERCC1 complex is a structure-specific endonuclease that plays an essential role in both NER and ICL repair^3–9^. Mutations in XPF-ERCC1 have been associated with several human inherited disorders, including Cockayne syndrome, xeroderma pigmentosum, cerebro-oculo-facio-skeletal syndrome, and Fanconi anemia^4,10–13^. As a nuclease, XPF‐ERCC1 is able to cleave a variety of DNA substrates, such as bubbles, flaps, and splayed arms^14–22^. Biochemical and structural studies have revealed that XPF and ERCC1 form a heterodimer in eukaryotic cells through the interaction of their conserved C-terminal tandem helix–hairpin–helix (HhH) motifs^23–26^. However, whether and how XPF binding to ERCC1 is regulated has not yet been established.

TIP60 is an acetyltransferase involved in the regulation of a wide variety of cellular activities, including gene transcription, chromatin remodeling, and DNA damage repair^27–29^. For instance, TIP60 directly acetylates ATM at Lys3016 to enhance ATM kinase activity, thereby regulating the cellular response to DSBs^30^. TIP60 has also been suggested to regulate the cellular response to ultraviolet (UV) irradiation, but the exact role of its acetyltransferase activity in this process remains largely unexplored^31–34^. Moreover, another study showed that TIP60 directly interacted with the Fanconi anemia protein FANCD2 and was critical for ICL repair^35^. However, downregulation of TIP60 did not affect FANCD2 mono-ubiquitination or its localization to DNA damage sites following treatment with mitomycin C (MMC)^35^, indicating that TIP60 may facilitate ICL repair by a yet-unknown mechanism.

In this study, we identify a regulatory mechanism underlying XPF-ERCC1 complex assembly and function by TIP60-dependent acetylation. We show that the XPF-ERCC1 interaction increases following UV irradiation or treatment with MMC. The damage-induced XPF-ERCC1 interaction requires TIP60-dependent acetylation of XPF at Lys911, which disrupts the Glu907-Lys911 salt bridge, thereby exposing a previously unidentified second binding site for ERCC1. Finally, we reveal that the damage-induced XPF-ERCC1 interaction is necessary for both NER and ICL repair. Our results provide mechanistic insight into the role of TIP60 in the maintenance of genome stability.

## Results

### TIP60 is required for efficient nucleotide excision repair

**Fig. 1:**
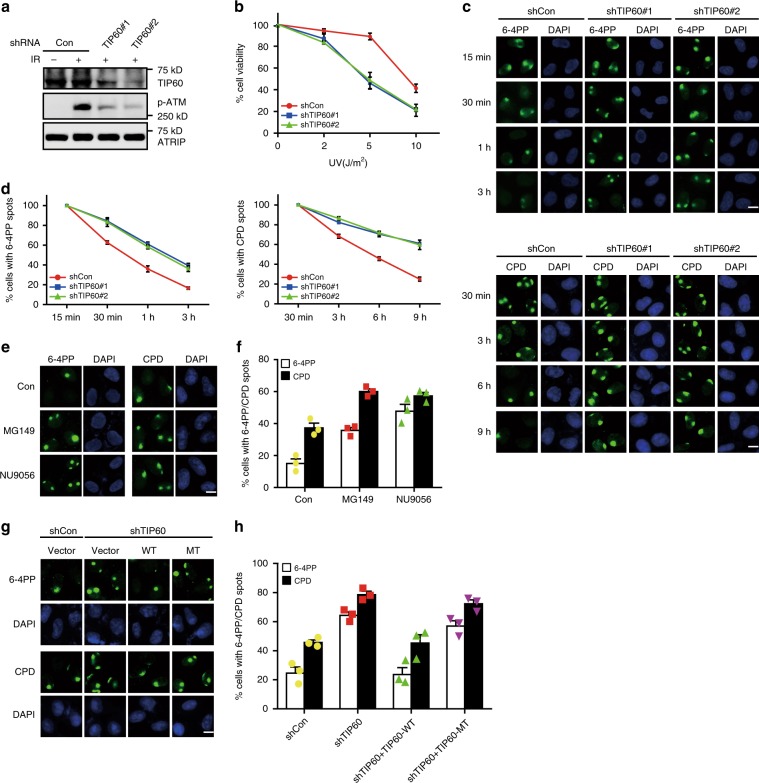
TIP60 is required for efficient NER. **a**, **b** HeLa cells were infected with lentiviral vectors expressing a control shRNA or TIP60-specific shRNAs and cultured in medium containing puromycin (2 μg ml^−1^). The resulting cells were then treated with 5 Gy IR or indicated doses of UV. TIP60 Knockdown efficiency was confirmed by immunoblotting (**a**). Clonogenic cell survival assays were performed in triplicates. Data represent means ± SEM from three independent experiments (**b**). **c**, **d** TIP60 is essential for the repair of UV-induced DNA lesions. HeLa cells were infected with lentiviral vectors expressing a control shRNA or TIP60-specific shRNAs and cultured in medium containing puromycin. The resulting cells were then irradiated with UV through 5-micron filters and allowed to recover for the indicated times before being stained with antibodies for 6-4PP or CPD (**c**). Data represent means ± SEM from three independent experiments (**d**). More than 1000 6-4PP or CPD spots were counted for each condition. **e**, **f** The acetyltransferase activity of TIP60 is essential for NER. HeLa cells were either mock treated with DMSO or treated with TIP60-specific inhibitors MG149 (100 μM) or NU9056 (20 μM) for 30 min before they were irradiated with UV through 5-micron filters and allowed to recover for 3 h (for 6-4PP) or 6 h (for CPD). Cells were then stained with antibodies for 6-4PP or CPD (**e**). Data represent means ± SEM from three independent experiments (**f**). More than 1000 6-4PP or CPD spots were counted for each condition. **g**, **h** A TIP60-depleted cell line stably expressing shRNA#2-resistant wild-type TIP60 or an acetyltransferase-defective mutant was generated. The resulting cells were irradiated with UV through 5-micron filters and allowed to recover for 3 h (for 6-4PP) or 6 h (for CPD) before being stained with antibodies for 6-4PP or CPD (**g**). Data represent means ± SEM from three independent experiments (**h**). More than 1000 6-4PP or CPD spots were counted for each condition. Scale bar, 10 μm. Source data are provided as a Source Data file.

To investigate the role of TIP60 in the repair of UV-induced DNA damage, we employed two short hairpin RNAs (shRNAs) to suppress endogenous TIP60 expression in HeLa cells. As previously reported, downregulation of TIP60 not only impairs ionizing radiation (IR)-induced ATM activation, but also causes hypersensitivity to the DNA crosslinking agent MMC (Fig. 1a and Supplementary Fig. 1A)^30,35^. Strikingly, TIP60 depletion also rendered cells more sensitive to UV irradiation, indicating that TIP60 might be involved in the repair of UV-induced lesions (Fig. 1b).

Translesion DNA synthesis (TLS) and NER are the two major DNA damage tolerance/repair pathways that confer cellular resistance to UV irradiation^36,37^. To investigate how TIP60 contributes to the tolerance/repair of UV damage, we first examined whether depletion of TIP60 affected UV-induced proliferating cell nuclear antigen (PCNA) mono-ubiquitylation, a central event in the TLS pathway^38^. As shown in Supplementary Fig. 1B, following UV irradiation, the levels of PCNA mono-ubiquitylation in TIP60-depleted cells were comparable to that in wild-type cells. In line with this, downregulation of TIP60 did not affect UV-induced polymerase eta (Polη) foci formation (Supplementary Fig. 1C, D). These findings suggest that TIP60 is not required for TLS and thus may play a role in the NER pathway.

Cyclobutane pyrimidine dimers (CPDs) and pyrimidine-(6–4)-pyrimidone photoproducts (6-4PPs) are two predominant types of UV-induced DNA lesions and are primarily repaired by the NER pathway^39^. We then compared the rates of repair of CPDs and 6-4PPs in UV-irradiated wild-type and TIP60-depleted cells at various time points post-irradiation. As shown in Fig. 1c, d and Supplementary Fig. 1E, F, compared with wild-type cells, TIP60-depleted cells displayed a much-delayed removal rate of CPDs and 6-4PPs, indicating that TIP60 is required for efficient NER. To determine whether the acetyltransferase activity of TIP60 is essential for its function in this process, we treated cells with MG149 or NU9056, two small-molecule inhibitors of TIP60. As shown in Fig. 1e, f and Supplementary Fig. 1G, H, inhibition of TIP60 acetyltransferase activity resulted in similar NER defects as TIP60 depletion. More importantly, TIP60-depleted cells expressing the acetyltransferase-inactivating mutant, Q377E/G380E (TIP60-MT), also exhibited much-delayed removal of CPDs and 6-4PPs compared with that of cells expressing wild-type TIP60 (TIP60-WT) (Fig. 1g, h and Supplementary Fig. 1I). Taken together, these results suggest that the acetyltransferase activity of TIP60 is indispensable for its function in NER.

### TIP60 interacts with and acetylates XPF

**Fig. 2:**
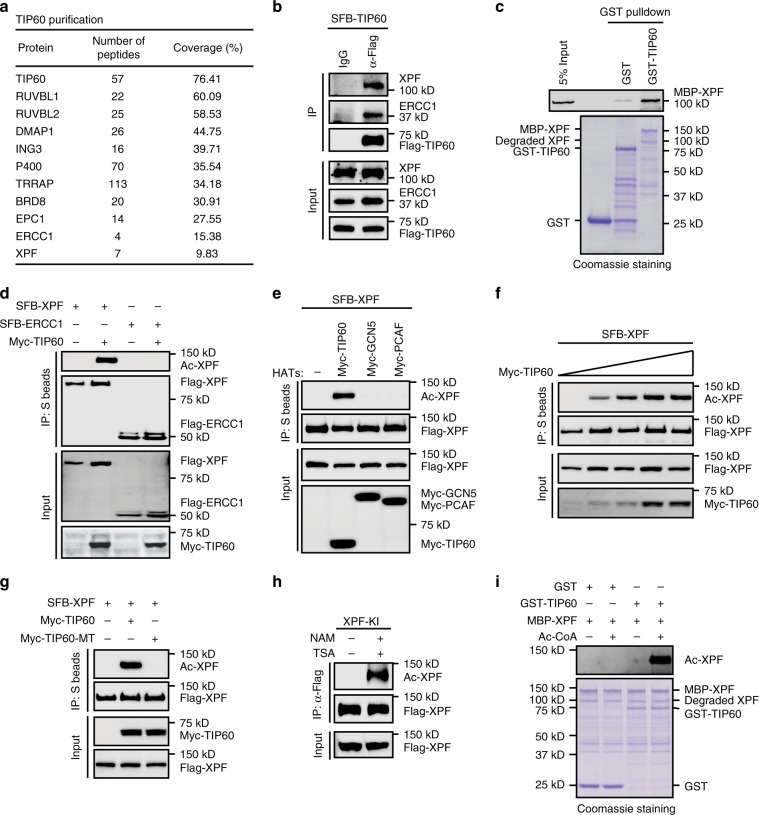
TIP60 interacts with and acetylates XPF. **a** Tandem affinity purification of TIP60 protein complexes. Proteins identified by Mass spectrometry analysis are listed. Bait protein is indicated in bold letters. **b** TIP60 forms a complex with XPF and ERCC1. Whole-cell lysates were prepared from HEK293T cells stably expressing SFB-tagged TIP60 and subjected to immunoprecipitation and western blot analysis was carried out as indicated. **c** TIP60 directly interacts with XPF in vitro. Upper panel: XPF was detected by immunoblotting. Lower panel: Proteins purified from *E. Coli* were resolved by SDS PAGE and visualized by Coomassie blue staining. **d**, **e** TIP60 acetylates XPF in vivo. Whole-cell lysates were prepared and subjected to immunoprecipitation with S beads, and western blot analysis was carried out as indicated. **f** HEK293T cells were transfected with plasmids encoding SFB-tagged XPF together with increasing amounts of plasmids encoding Myc-tagged TIP60 (0.5 μg, 1 μg, 2 μg, 4 μg) for 24 h. Whole-cell lysates were then prepared and subjected to immunoprecipitation with S beads and western blot analysis was carried out as indicated. **g** HEK293T cells were transfected with the indicated plasmids for 24 h. Whole-cell lysates were then prepared and subjected to immunoprecipitation with S beads and western blot analysis was carried out as indicated. **h** XPF-SFB knock-in HeLa cells were either untreated or treated with Nicotinamide (10 mM) and TSA (10 μM) for 4 h. Whole-cell lysates were then incubated with protein A agarose beads conjugated with anti-Flag antibody, and western blot analysis was carried out as indicated. **i** TIP60 acetylates XPF in vitro. MBP-tagged XPF, GST, or GST-tagged TIP60 were purified from *E. coli*. The purified proteins were then incubated in reaction buffer in the presence or absence of the acetyl-CoA (2 mM) at 37 °C for 30 min. Upper panel: XPF acetylation was detected by immunoblotting. Lower panel: Proteins purified from *E. Coli* were resolved by SDS PAGE and visualized by Coomassie blue staining. Source data are provided as a Source Data file.

To gain insight into how TIP60 participates in the NER pathway, we purified proteins interacting with TIP60 using tandem affinity purification. In addition to these known TIP60-interacting proteins such as RUVBL1, RUVBL2, and DMAP1, we also identified several protein complexes associated with the DNA damage response (Supplementary Data 1). Notably, one of these complexes, the XPF-ERCC1 complex is related to the NER pathway (Fig. 2a and Supplementary Data 1). The presence of XPF and ERCC1 in TIP60 purification was further confirmed by western blots (Fig. 2b). Moreover, in vitro GST pull-down assays with recombinant GST-tagged TIP60 and MBP-tagged XPF or ERCC1 purified from *E. coli* demonstrated that TIP60 directly interacted with XPF but not ERCC1 (Fig. 2c and Supplementary Fig. 2A, B). These results suggest that TIP60 associates with the XPF-ERCC1 complex primarily through XPF.

To explore whether XPF and/or ERCC1 could be the substrate(s) for the acetyltransferase TIP60, HEK293T cells were co-transfected with Myc-tagged TIP60 together with SFB-tagged XPF or ERCC1. Cell lysates were then subjected to pull-down assays with S protein beads and immunoblotted with a pan-anti-acetyl-lysine antibody. As shown in Fig. 2d, XPF, but not ERCC1, was efficiently acetylated by TIP60. By contrast, GCN5 and PCAF, were unable to acetylate XPF in similar assays, demonstrating the specificity of TIP60 in XPF acetylation (Fig. 2e). Furthermore, TIP60 acetylated XPF in a dose-dependent manner (Fig. 2f). More importantly, the enzymatically inactive mutant of TIP60 failed to acetylate XPF (Fig. 2g).

We next wanted to assess whether endogenous XPF could be acetylated. Since our homemade anti-XPF antibody was unable to precipitate the endogenous XPF protein, we fused an SFB tag onto the C-terminus of the endogenous XPF gene using the recombinant adeno-associated virus-based knock-in approach^40,41^. With this method, the endogenous XPF protein can be recognized by the anti-Flag antibody. We thus incubated the lysates derived from XPF-SFB knock-in HeLa cells with anti-Flag antibody and immunoblotted the resulting immunoprecipitates with the pan anti-acetyl-lysine antibody. As shown in Fig. 2h, acetylation of endogenous XPF was clearly detected in cells treated with the deacetylase inhibitors trichostatin A (TSA) and nicotinamide (NAM), but not in untreated control cells.

We further used the purified *E. coli*-expressed MBP-tagged XPF and GST-tagged TIP60 to perform in vitro acetylation assays. As shown in Fig. 2i, XPF was acetylated by TIP60, but not by the GST tag, in vitro.

### XPF is acetylated at lysine 911

**Fig. 3:**
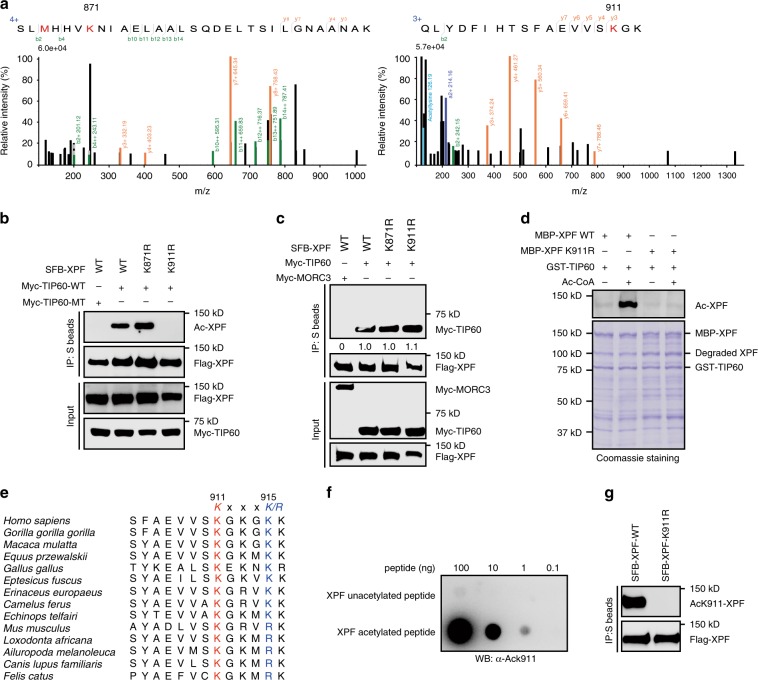
TIP60 acetylates XPF at lysine 911. **a** XPF acetylation was analyzed by mass spectrometry. HEK293T cells stably expressing SFB-tagged XPF were treated with Nicotinamide (10 mM) and TSA (10 μM) for 4 h. SFB-XPF was then purified and subjected to MS analysis (the mass tolerance is 20 ppm). **b** TIP60 acetylates XPF at K911 in vivo. HEK293T cells were transiently transfected with the indicated plasmids for 24 h. Whole-cell lysates were then prepared and subjected to immunoprecipitation with S beads and western blot analysis was carried out as indicated. **c** HEK293T cells were transiently transfected with the indicated plasmids for 24 h. Whole-cell lysates were then immunoprecipitated with S beads, and western blot analysis was carried out as indicated. The amount of co-immunoprecipitated TIP60 was quantified by ImageJ, normalized to total immunoprecipitated XPF, and shown below the blot. **d** TIP60 acetylates XPF at K911 in vitro. Wild-type XPF or the K911R mutant purified from *E. Coli* were incubated with recombinant TIP60 protein in reaction buffer in the presence or absence of the acetyl-CoA (2 mM) at 37 °C for 30 min. Upper panel: XPF acetylation was detected by immunoblotting. Lower panel: Purified proteins were resolved by SDS-PAGE and visualized by Coomassie blue staining. **e** Sequence alignment of the region containing the acetylation site in XPF from different species. **f** Characterization of the anti-AcK911-XPF antibody by a dot blot assay. Various amounts of unacetylated or acetylated XPF-K911 peptides were spotted onto nitrocellulose membrane and immunoblotted with the anti-AcK911-XPF antibody. **g** HEK293T cells were transfected with the indicated plasmids for 24 h. Whole-cell lysates were then incubated with S beads and western blot analysis was carried out as indicated. Source data are provided as a Source Data file.

To identify the potential acetylation site(s) on XPF, we purified stably expressed XPF from HEK293T cells pretreated with the deacetylase inhibitors TSA and NAM. Mass spectrometry analysis revealed two lysine residues (Lys871 and Lys911) as candidate acetylation sites on XPF (Fig. 3a). We therefore mutated each lysine residue into arginine (K871R or K911R) to test which residue was acetylated. As shown in Fig. 3b, the K911R mutant, but not the K871R mutant, completely lost its ability to be acetylated by TIP60. Moreover, the K911R mutant interacted with TIP60 as efficiently as wild-type XPF and the K871R mutant (Fig. 3c). To further confirm the specific acetylation at Lys911 by TIP60, we carried out an in vitro acetylation assay using the purified K911R mutant. As shown in Fig. 3d, wild-type XPF, but not the K911R mutant, was acetylated by TIP60 in vitro. These results suggest that Lys911 is the major acetylation site of XPF. Of note, Lys911 of XPF is embedded in an acetylation consensus sequence KXXXK/R (residues 911–915), and is evolutionarily conserved (Fig. 3e). To date, mutation of XPF at Lys911 has not been reported in XP-F patients. We further generated an antibody specific to acetylated Lys911 and verified its specificity by dot blot assays (Fig. 3f). As expected, the site-specific AcK911-XPF antibody recognized wild-type XPF but not the K911R mutant (Fig. 3g).

### SIRT1 interacts with and deacetylates XPF

**Fig. 4:**
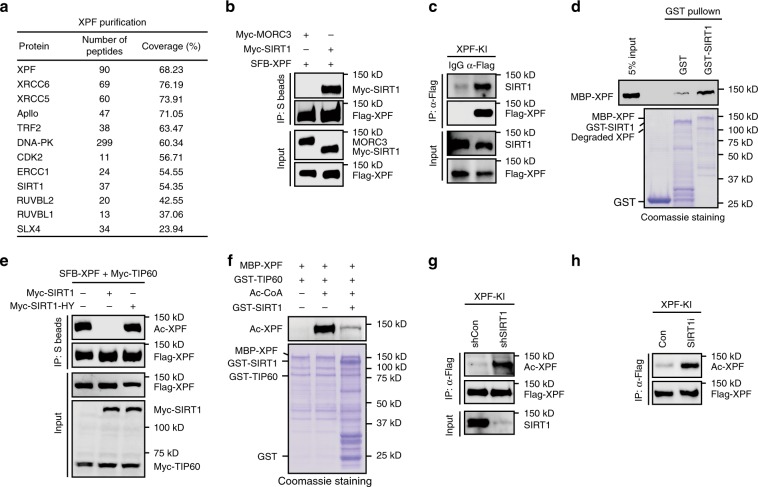
SIRT1 interacts with and deacetylates XPF. **a** Tandem affinity purification of XPF protein complexes from HEK293T cells stably expressing SFB-tagged XPF. Proteins identified by Mass spectrometry analysis are listed. Bait protein is indicated in bold letters. **b** SIRT1 interacts with XPF. HEK293T cells were transiently transfected with the indicated plasmids for 24 h. Whole-cell lysates were then prepared and subjected to immunoprecipitation with S beads and western blot analysis was carried out as indicated. **c** Association of endogenous SIRT1 with XPF. Whole-cell lysates prepared from XPF-SFB knock-in HeLa cells were incubated with protein A agarose beads conjugated with anti-Flag antibody and western blot analysis was carried out as indicated. **d** SIRT1 directly interacts with XPF in vitro. Upper panel: XPF was detected by immunoblotting. Lower panel: Purified proteins were resolved by SDS-PAGE and visualized by Coomassie blue staining. **e** SIRT1 mediates XPF deacetylation. HEK293T cells were transfected with the indicated plasmids for 24 h. Whole-cell lysates were then prepared and subjected to immunoprecipitation with S beads and western blot analysis was carried out as indicated. **f** SIRT1 deacetylates XPF in vitro. XPF proteins purified from *E. Coli* were acetylated by recombinant TIP60 proteins in vitro. Subsequently, acetylated XPF, as a substrate, was incubated with recombinant SIRT1 protein at 37 °C for 30 min. The reaction mixtures were then subjected to SDS PAGE and immunoblotted with anti-acetyl lysine antibody (Upper panel). Purified proteins were resolved by SDS-PAGE and visualized by Coomassie blue staining (Lower panel). **g**, **h** SIRT1 depletion or inhibition increases XPF acetylation. XPF-SFB knock-in HeLa cells were infected with SIRT1-specific lentiviral shRNAs or were treated with SIRT1 inhibitor EX527 (20 μM) for 6 h. Whole-cell lysates were then incubated with protein A agarose beads conjugated with anti-Flag antibody and western blot analysis was carried out as indicated. Source data are provided as a Source Data file.

Lysine acetylation is a highly dynamic and reversible process. To identify the specific deacetylase(s) responsible for XPF deacetylation, we generated a HEK293T cell line stably expressing SFB-tagged XPF and performed tandem affinity purification to identify XPF-associated proteins. In addition to known XPF-associated proteins such as ERCC1 and SLX4, we also found that the deacetylase SIRT1 existed in XPF protein complexes (Fig. 4a and Supplementary Data 2). We thereby confirmed the SIRT1-XPF interaction by co-immunoprecipitation experiments. As shown in Fig. 4b, c, SIRT1 interacted with XPF in both endogenous and overexpression systems. We further performed in vitro GST pull-down assays and found that XPF interacted with SIRT1 directly (Fig. 4d). The direct interaction of XPF and SIRT1 prompted us to examine whether SIRT1 has a direct role in the regulation of XPF deacetylation. We co-transfected SIRT1 together with XPF and TIP60 for use in the acetylation assay. As shown in Fig. 4e, overexpression of wild-type SIRT1, but not the catalytic-inactive H363Y mutant (SIRT-HY), resulted in the deacetylation of XPF in cells. We further tested whether SIRT1 could directly deacetylate XPF in vitro. XPF was first acetylated in the in vitro acetylation assay using the recombinant protein. Acetylated XPF was then used as a substrate in the SIRT1 deacetylation assay. As shown in Fig. 4f, XPF was efficiently deacetylated by recombinant SIRT1 in vitro. In addition, depletion of SIRT1 by specific shRNA or inhibition of SIRT1 activity by chemical inhibitor led to a dramatic increase in the acetylation of endogenous XPF (Fig. 4g, h). More importantly, other nuclear localized sirtuins including SIRT2, SIRT6, and SIRT7 failed to deacetylate XPF (Supplementary Fig. 3). Together, these results suggest that SIRT1 is a bona fide XPF-interacting protein and mediates XPF deacetylation.

### XPF acetylation increases in response to UV and MMC

**Fig. 5:**
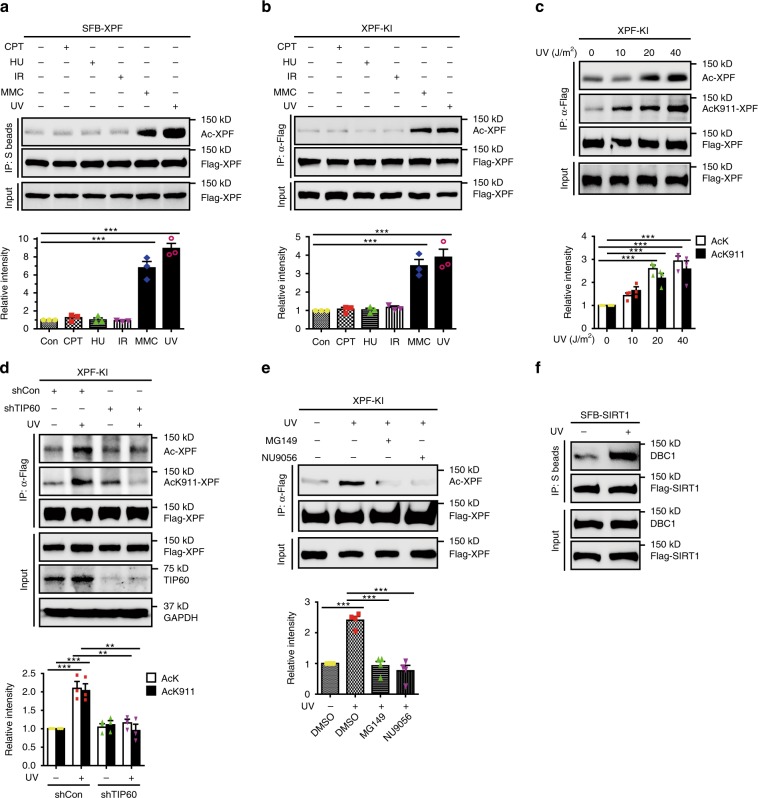
UV or MMC stimulates XPF acetylation. **a** HEK293T cells expressing SFB-tagged XPF were treated with 1 μM CPT, 5 mM HU, 10 Gy IR, or 40 J m^−2^ UV for 4 h, or 1 μM MMC for 12 h. The levels of Ac-XPF were quantified by ImageJ and normalized to total immunoprecipitated XPF. Data represent means ± SEM from three independent experiments. ***P < 0.001, One-way ANOVA with Dunnett’s Multiple Comparison test. **b** XPF-SFB knock-in HeLa cells were treated with 1 μM CPT, 5 mM HU, 10 Gy IR, or 40 J/m^2^ UV for 4 h, or 1 μM MMC for 12 h. The levels of Ac-XPF were quantified by ImageJ and normalized to total immunoprecipitated XPF. Data represent means ± SEM from three independent experiments. ***P < 0.001, One-way ANOVA with Dunnett’s Multiple Comparison test. **c** XPF-SFB knock-in HeLa cells were treated with UV irradiation and harvested 4 h later. The levels of Ac-XPF were quantified by ImageJ and normalized to total immunoprecipitated XPF. Data represent means ± SEM from three independent experiments. ***P < 0.001, Two-way ANOVA with Bonferroni post-tests. **d**, **e** TIP60 is required for UV-induced XPF acetylation. XPF-SFB knock-in HeLa cells were infected with TIP60-specific lentiviral shRNAs (**d**) or were treated with TIP60 inhibitors MG149 (100 μM) or NU9056 (20 μM) for 30 min (**e**) before they were irradiated with 40 J m^−2^ UV and allowed to recover for 4 h. The levels of Ac-XPF were quantified by ImageJ and normalized to total immunoprecipitated XPF. Data represent means ± SEM from three independent experiments. **P < 0.01, ***P < 0.001, Two-way ANOVA with Bonferroni post-tests or One-way ANOVA with Dunnett’s Multiple Comparison test. **f** DBC1-SIRT1 interaction increases following UV irradiation. HEK293T cells transfected with SFB-SIRT1 were treated with 40 J m^−2^ UV and allowed to recover for 2 h. Whole-cell lysates were subjected to immunoprecipitation with S beads and western blot analysis was carried out as indicated. Source data are provided as a Source Data file.

We next examined whether the acetylation of XPF can be regulated by DNA damage. As shown in Fig. 5a, b, both UV irradiation and MMC treatment caused a dramatic increase in the acetylation levels of ectopically expressed and endogenous XPF. By contrast, the levels of acetylated XPF were not obviously affected by IR irradiation, the DNA topoisomerase I inhibitor camptothecin, or the DNA replication inhibitor hydroxyurea. Furthermore, UV irradiation and MMC treatment increased XPF acetylation in a dose-dependent manner (Fig. 5c and Supplementary Fig. 4A). Interestingly, UV-induced XPF acetylation at lysine 911 only occurs on a small fraction (about 25%) of total XPF (Supplementary Fig. 4B).

To determine whether UV-induced XPF acetylation was dependent on the acetyltransferase TIP60, we downregulated TIP60 expression in cells by TIP60-specific shRNA. As shown in Fig. 5d, depletion of TIP60 significantly prevented UV-induced XPF acetylation. Similar results were obtained when cells were treated with the TIP60-specific inhibitors MG149 or NU9056 (Fig. 5e).

Given that XPF interacted with TIP60 and SIRT1 directly and UV irradiation stimulated acetylation of XPF, we speculated that UV irradiation may alter the interaction of XPF with TIP60 or SIRT1. Surprisingly, the interaction of XPF and TIP60 or SIRT1 was unaffected following UV irradiation (Supplementary Fig. 4C, D), indicating that other mechanisms may exist.

Previous studies have shown that Deleted in Breast Cancer-1 (DBC1) interacts with SIRT1 directly and thus inhibits its deacetylase activity^42,43^. Interestingly, genotoxic stress, including IR, etoposide, and H2O2, was able to enhance the SIRT1-DBC1 interaction^44^. We thus examined whether UV irradiation or MMC treatment could also modulate the SIRT1-DBC1 interaction, thereby inhibiting SIRT1 activity and increasing XPF acetylation. Indeed, following UV irradiation or MMC treatment, DBC1 bound more tightly to SIRT1 (Fig. 5f and Supplementary Fig. 4E).

### XPF acetylation facilitates XPF-ERCC1 complex assembly

**Fig. 6:**
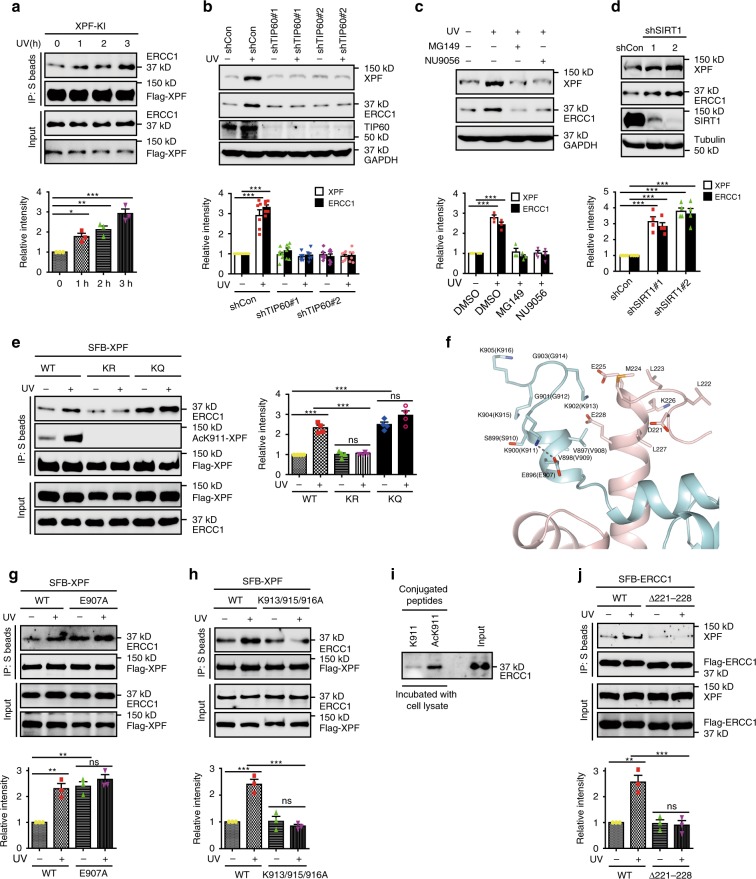
XPF acetylation facilitates XPF-ERCC1 complex assembly. **a** XPF-SFB knock-in cells were lysed and subjected to immunoprecipitation. Lysates were adjusted on the basis of XPF and ERCC1 protein levels. The amount of co-immunoprecipitated ERCC1 was quantified by ImageJ and normalized to immunoprecipitated XPF. Data represent means ± SEM from three independent experiments. *P < 0.05, **P < 0.01, ***P < 0.001, One-way ANOVA with Dunnett’s Multiple Comparison test. **b** XPF and ERCC1 levels were quantified by ImageJ. Data represent means ± SEM from three independent experiments. ***P < 0.001, Two-way ANOVA with Bonferroni post-tests. **c**, **d** HeLa cells were treated with MG149 (100 μM) or NU9056 (20 μM) or infected with SIRT1 shRNAs. Data represent means ± SEM from three independent experiments. ***P < 0.001, Two-way ANOVA with Bonferroni post-tests. **e** The amount of co-immunoprecipitated ERCC1 was quantified by ImageJ and normalized to immunoprecipitated XPF. Data represent means ± SEM from three independent experiments. ns not significant, ***P < 0.001, One-way ANOVA with Bonferroni’s Multiple Comparison test. **f** Arrangement of XPF-ERCC1 residues near Lys911 based on NMR structure (PDB:1z00, model01). XPF is shown in cyan, ERCC1 is shown in pink, gray dashed lines indicate the distance (2.8 Å) between the ε-nitrogen atom in Lys911 and the carbonyl oxygen atom in Glu907. The numbering in the NMR structure is off by 11 compared with the sequence used in this study (shown in parentheses). Figure was generated by the PyMOL Molecular Graphics System (Version 2.1.0 Open-Source) (Http://www.pymol.org). **g**, **h** The amount of co-immunoprecipitated ERCC1 was quantified by ImageJ and normalized to immunoprecipitated XPF. Data represent means ± SEM from three independent experiments. ns not significant, **P < 0.01, ***P < 0.001, One-way ANOVA with Bonferroni’s Multiple Comparison test. **i** Nonacetylated or acetylated peptides were conjugated to Sepharose beads and incubated with HEK293T cell lysates. **j** The amount of co-immunoprecipitated XPF was quantified by ImageJ and normalized to immunoprecipitated ERCC1. Data represent means ± SEM from three independent experiments. ns not significant, **P < 0.01, ***P < 0.001, One-way ANOVA with Bonferroni’s Multiple Comparison test. Source data are provided as a Source Data file.

It has been well established that XPF and ERCC1 form a heterodimer through the interaction of their conserved C-terminal tandem HhH motifs^23–26^. Since Lys911 is highly conserved among species and is located close to the HhH motifs of XPF, we hypothesized that TIP60-mediated acetylation of XPF may affect XPF-ERCC1 complex assembly. To test this, we first examined whether the XPF-ERCC1 interaction is regulated by UV irradiation or MMC treatment. As shown in Fig. 6a and Supplementary Fig. 5A, the XPF-ERCC1 interaction increased following UV irradiation or MMC treatment. Interestingly, and consistent with the observation that formation of the XPF-ERCC1 heterodimer is essential for the stability of both proteins, the protein levels of both XPF and ERCC1 increased following UV irradiation or MMC treatment (Supplementary Fig. 5B, C). Moreover, TIP60 depletion or inhibition of TIP60 acetyltransferase activity abolished the increase in XPF and ERCC1 protein levels induced by UV irradiation (Fig. 6b, c). Conversely, SIRT1 depletion or inhibition of SIRT1 deacetylase activity resulted in a dramatic increase in the protein levels of XPF and ERCC1 even in the absence of DNA damage (Fig. 6d and Supplementary Fig. 5D). Importantly, downregulation of TIP60 or SIRT1 had no effect on the mRNA levels of both XPF and ERCC1 (Supplementary Fig. 5E, F).

We next determined whether acetylation of XPF is required for the damage-induced interaction of XPF and ERCC1. As shown in Fig. 6e, loss of Lys911 acetylation of XPF abolished the UV-induced XPF-ERCC1 interaction without affecting the constitutive interaction. In addition, the acetylation-mimic mutant K911Q interacted with ERCC1 to a greater extent than wild-type XPF in both the absence and presence of UV damage (Fig. 6e). In contrast, substitution of a nearby lysine residue Lys913, Lys915, or Lys916 with Gln (K913Q, K915Q, or K916Q) did not show obviously enhanced binding to ERCC1 (Supplementary Fig. 5G). Furthermore, in our western blotting experiments, we routinely found that the expression levels of the K911R mutant were much lower than that of wild-type XPF and the K911Q mutant (Supplementary Fig. 5H). These results suggest that TIP60-mediated acetylation of XPF at Lys911 facilitates XPF-ERCC1 complex assembly. Consistent with this conclusion, knockdown of TIP60 reduced the recruitment of XPF to UV-induced DNA damage sites (Supplementary Fig. 5I). In contrast, TIP60 depletion had no effect on the recruitment of the upstream NER factors including XPA, XPB, and XPC to UV lesions^8^ (Supplementary Fig. 5I).

### XPF acetylation exposes a second binding site for ERCC1

In addition to increasing the hydrophobicity of the lysine side chain, lysine acetylation often induces a conformational change by neutralizing the positive charge of the amino acid. Interestingly, in the nuclear magnetic resonance structure (PDB:1z00)^23^, Lys911 of XPF is positioned in the immediate vicinity of Glu907, indicating a direct salt bridge between the positively charged Lys911 and the negatively charged Glu907 (Fig. 6f). More strikingly, structural analysis revealed that the corresponding region in ERCC1 (amino acids 221–228) is mostly composed of negatively charged as well as hydrophobic residues, which complement well with the positively charged and hydrophobic residues surrounding acetylated Lys911 (amino acids 911–916) (Fig. 6f). Given that acetylation of XPF enhances its interaction with ERCC1, we hypothesized that this acetylation may eliminate the positive charge of Lys911 and disrupt the electrostatic interaction with Glu907, thereby exposing a second binding site for ERCC1. To test this hypothesis, we first mutated the negatively charged glutamic acid residue to alanine (E907A) in XPF and examined its interaction with ERCC1. Similar to the K911Q mutant, the XPF E907A mutant also exhibited enhanced interaction with ERCC1 regardless of UV irradiation (Fig. 6g). Furthermore, substitutions of the positively charged residues (K913A, K915A, and K916A) with neutral uncharged residues in XPF abolished the UV-induced interaction of XPF and ERCC1 (Fig. 6h). More importantly, the acetylated Lys911 peptide (Ac-K911) alone, but not the nonacetylated Lys911 peptide (K911), was able to pull-down endogenous ERCC1 in the absence of UV damage (Fig. 6i). These results suggest that acetylated Lys911 and its surrounding residues in XPF provide a second binding site for ERCC1.

Next, we examined whether the region in ERCC1 (amino acids 221–228) is essential for the UV-induced XPF-ERCC1 interaction. We generated an ERCC1 deletion mutant lacking this region and performed co-immunoprecipitation experiments. As shown in Fig. 6j, deletion of this region abolished the UV-induced XPF-ERCC1 interaction. Consistently, substitutions of the negatively charged residues (D221A, E225A, and E228A) or hydrophobic residues (L223A, M224A, and L227A) with neutral uncharged residues in this region of ERCC1 also abolished the UV-induced XPF-ERCC1 interaction (Supplementary Fig. 5J). Given that mutation at K913, but not K915 or K916 in XPF, diminished UV-induced XPF-ERCC1 interaction (Supplementary Fig. 5G), we speculate that the Lys913 residue of XPF is critical for its interaction with this region in ERCC1. Together these results suggested that, while the HhH motifs present in both proteins mediate the constitutive XPF-ERCC1 interaction, acetylated Lys911 and the surrounding residues in XPF and the region in ERCC1 (amino acids 221–228) contribute to their stress-induced interaction.

### XPF acetylation is critical for NER and ICL repair

**Fig. 7:**
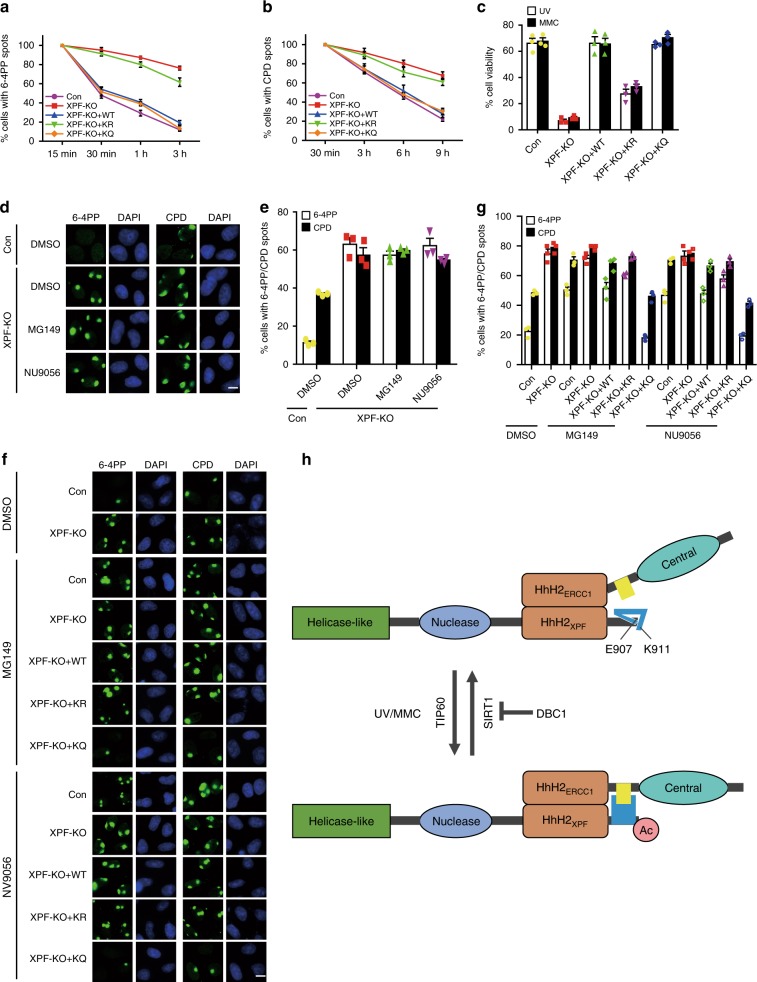
XPF acetylation promotes NER and ICL repair. **a**, **b** XPF-deficient cells transfected with the indicated plasmids were irradiated with UV through 5-micron filters and allowed to recover for the indicated times before being stained with antibodies for 6-4PP or CPD. Data represent means ± SEM from three independent experiments. More than 1000 6-4PP or CPD spots were counted for each condition. The amounts of indicated plasmids were adjusted to ensure comparable protein levels. **c** XPF-deficient cells transfected with the indicated plasmids were treated with UV irradiation (5 J m^−2^) or MMC (40 nM, 24 h). Experiments were performed in triplicates and results were presented as means ± SEM. The amounts of indicated plasmids were adjusted to ensure comparable protein levels. **d**, **e** XPF-deficient cells were treated with MG149 (100 μM) or NU9056 (20 μM) for 30 min before they were irradiated with UV through 5-micron filters and allowed to recover for 3 h (for 6-4PP) or 6 h (for CPD). Cells were then stained with antibodies for 6-4PP or CPD. Data represent means ± SEM from three independent experiments. More than 1000 6-4PP or CPD spots were counted for each condition. **f**, **g** XPF-deficient cells transfected with the indicated plasmids were treated with MG149 (100 μM) or NU9056 (20 μM) for 30 min before they were irradiated with UV through 5-micron filters and allowed to recover for 3 h (for 6-4PP) or 6 h (for CPD). Cells were then stained with antibodies for 6-4PP or CPD. Data represent means ± SEM from three independent experiments. More than 1000 6-4PP or CPD spots were counted for each condition. The amounts of plasmids expressing wild-type XPF, the K911R mutant, or the K911Q mutant for transfection were adjusted to ensure comparable protein levels. **h** A model of the role for XPF acetylation in the NER and ICL pathway. Scale bar, 10 μm. Source data are provided as a Source Data file.

To investigate whether XPF acetylation is required for NER, we knocked out XPF in HeLa cells by CRISPR/Cas9. As expected, XPF-deficient cells exhibited hypersensitivity to UV irradiation and a much slower removal rate of both CPDs and 6-4PPs when compared with wild-type cells (Fig. 7a–c and Supplementary Fig. 6A–D). Notably, the defects caused by loss of XPF were largely reversed by re-expression of wild-type XPF or the acetylation-mimic mutant K911Q, but not by the acetylation-defective mutant K911R, indicating that XPF acetylation is critical for efficient removal of UV-induced DNA lesions (Fig. 7a–c and Supplementary Fig. 6A–D). In addition, inhibition of TIP60 in XPF-deficient cells did not show any additional defects in the repair of CPDs and 6-4PPs, suggesting that XPF and TIP60 operate in the same pathway (Fig. 7d, e and Supplementary Fig. 6E). More strikingly, in XPF-deficient cells, expression of the K911Q mutant, but not wild-type XPF or the K911R mutant, was able to largely reverse the NER defects caused by TIP60 inhibition (Fig. 7f, g and Supplementary Fig. 6F). These results suggest that TIP60 promotes NER by directly acetylating XPF.

In addition to its essential role in NER, the XPF-ERCC1 complex has also been implicated in the repair of interstrand crosslink damage. We therefore tested whether XPF acetylation is important for ICL repair. As shown in Fig. 7c, knockout of XPF led to hypersensitivity to MMC treatment as expected. Interestingly, re-expression of wild-type XPF, but not the K911R mutant, was able to reverse the defects in XPF-deficient cells (Fig. 7c). More importantly, similar to wild-type XPF, the K911Q mutant was also able to reverse the defects caused by XPF deficiency, indicating that TIP60-mediated acetylation of XPF at Lys911 is critical for ICL repair (Fig. 7c).

## Discussion

Despite the importance of TIP60 in the cellular response to UV irradiation and ICL damage, little is known about the underlying mechanism. In this study, we provide evidence that TIP60 plays an essential role in both NER and ICL repair pathways. We show that TIP60 directly interacts with and acetylates XPF at Lys911 following both UV irradiation and MMC treatment. We further show that this acetylation is essential for UV- or MMC-induced XPF-ERCC1 interaction and the subsequent full assembly and activation of the complex. Given the central role of the XPF-ERCC1 complex in NER and ICL repair pathways, it is not surprising that TIP60-deficient cells are hypersensitive to UV irradiation and MMC treatment. Our results offer a previously uncharacterized molecular mechanism underlying the tumor suppressor activity of the acetyltransferase TIP60. Notably, besides XPF, many other proteins can also be acetylated by TIP60, including ATM, p53, and histone proteins. Thus, it is not surprising that TIP60 has various XPF-ERCC1-independent functions. For example, while XPF- or ERCC1-null mice are viable but show a severe postnatal growth defect, mice deficient for TIP60 are early embryonic lethal.

In addition to NER and ICL repair, the XPF-ERCC1 complex is suggested to play a critical role in the regulation of chromatin looping, gene expression, as well as CTCF-mediated genome organization^45,46^. Given that TIP60 facilitates the XPF-ERCC1 complex assembly and activation, it would therefore be worthwhile to investigate whether TIP60 is also involved in regulating these processes in the future studies. Protein lysine acetylation is a reversible and dynamic post-translational modification. In the present study, we show that acetylation of XPF is reversibly controlled by the deacetylase SIRT1. SIRT1 directly interacts with and deacetylates XPF both in vitro and in cells. In addition to XPF, SIRT1 has also been shown to deacetylate and interact with another key NER factor XPA^47^. These results suggest that SIRT1 has multiple roles in NER. Of note, a previous study revealed that SIRT1 activity could be negatively regulated by genotoxic stress, such as IR, etoposide, and H_2_O_2_^44^. Mechanistically, ATM kinase, activated by DNA damage, directly phosphorylates DBC1 at Thr454, a widely known negative regulator of SIRT1, to enhance its interaction with SIRT1, resulting in the inhibition of SIRT1 activity^44^. Strikingly, we found that, following UV irradiation or MMC treatment, DBC1 also binds more tightly to SIRT1, indicating that the stress-induced SIRT1-DBC1 interaction is a general mechanism for restraining the activity of SIRT1 in response to genotoxic stress. It was previously described that autoacetylation of TIP60 in response to UV damage is critical for its activation and that this activation is negatively regulated by SIRT1^48^. Thus, inhibition of SIRT1 activity by genotoxic stress has both direct (SIRT1 as a deacetylase for XPF) and indirect (SIRT1 as a deacetylase for TIP60) roles in the regulation of XPF acetylation. Based on these results, we propose the following working model. In the absence of DNA damage, SIRT1 physically interacts with XPF and maintains XPF in the nonacetylated state. Nonacetylated XPF interacts with ERCC1 solely via its C-terminal HhH motifs due to the formation of a salt bridge between Glu907 and Lys911, leading to incomplete assembly of the XPF-ERCC1 protein complex. Upon UV irradiation or MMC treatment, activated ATM kinase phosphorylates DBC1 at Thr454 to enhance its binding to SIRT1, which in turn inhibits SIRT1 activity and thereby contributes to XPF acetylation. Once acetylated, the salt bridge between Glu907 and Lys911 is disrupted, resulting in exposure of a second binding site for ERCC1 and subsequent full assembly and activation of XPF-ERCC1 (Fig. 7h). Our findings have uncovered a mechanism underlying XPF-ERCC1 complex assembly and activation in response to DNA damage.

While UV irradiation or MMC treatment is able to trigger a strong dose-dependent induction of XPF acetylation, other DNA-damaging agents, such as IR, fail to do so. However, upon exposure to IR, TIP60 is activated. IR can also induce the interaction between SIRT1 and its inhibitor DBC1. These results indicate that, in addition to activating TIP60 and inhibiting SIRT1, there are likely other mechanisms involved in the regulation of acetylation levels of XPF following UV irradiation or MMC treatment.

## Methods

### Antibodies

Anti-XPF (1:1000) polyclonal antibody was generated by immunizing rabbits with MBP-XPF (residues 616–916) fusion proteins purified from *E. Coli* (HuaAn Biotechnology, Hangzhou, China). Antisera were affinity-purified using AminoLink plus Immobilization and purification kit (Pierce). Anti-CPD (NM-DND-001, 1:600) and anti-(6–4)PP (NM-DND-002, 1:600) antibodies were purchased from MBL International Corporation. Anti-TIP60 (ARG65853, 1:500) antibody and anti-acetyl lysine (9441S, 1:1000) antibodies were purchased from Arigo Biolaboratories and Cell Signaling Technology, respectively. Anti-phospho-ATM (S1981) (ab81292, 1:1000), anti-ERCC1 (ab129267, 1:2,000), anti-ATRIP (ab175221, 1:500), and anti-SIRT1 (ab32441, 1:1000) antibodies were purchased from Abcam. Anti-Myc (9E10, 1:1000) and anti-Flag (Clone M2, 1:10,000) antibodies were purchased from Covance and Sigma, respectively. Anti-GAPDH (MAB374, 1:3000) and anti-H3 (04–928, 1:500) antibody was purchased from Millipore. Anti-DBC1 (A303-942A, 1:1000) and anti-PCNA (SC-56, 1:500) antibodies were purchased from Bethyl Laboratories and Santa Cruz, respectively. The site-specific AcK911-XPF (1:300) antibody was generated using the following peptide: aa 905–916 FAEVVSK (Ac)GKGKK.

### Cell culture and shRNAs

HeLa and HEK293T cells were purchased from ATCC and grown in DMEM (Gibco) supplemented with 10% fetal bovine serum and 1% penicillin and streptom ycin (Life Technologies) at 37 °C in 5% CO_2_ atmosphere. Lentivirus-based shRNAs were purchased from Thermo Fisher. The shRNA nontarget sequence is: 5′-CCCATAAGAGTAATAATAT-3′; The TIP60 targeting sequences are: #1, 5′-CCTTGACCATAAGACACTGTA-3′; 2#, 5′-CCTCCTATCCTATCGAAGCTA-3′; The SIRT1 targeting sequences are: 1#, 5′-CAGGTCAAGGGATGGTATTTA-3′; 2#, 5′-CATGAAGTGCCTCAGATATTA-3′. The shRNA-resistant wild-type and mutant TIP60 constructs were generated by changing seven nucleotides in the shRNA#2 targeting region (C1230A, C1231T, C1233A, A1236G, C1239A, T1242C, C1243A, A1245G, A1246T, G1247C, and C1248A substitutions). Lentiviruses were generated in HEK293T cells. Briefly, the shRNA plasmids were co-transfected with the packaging plasmids pMD2G and pSPAX2 into HEK293T cells. After 48 h of transfection, the viral supernatant was used to infect HeLa cells. Infected HeLa cells were then selected with media containing puromycin (2 μg ml^−1^, Sigma).

### CRISPR/Cas9 knockout

The following guide RNAs were used to generate XPF knockout HeLa cells: XPF#1: GCCATGGCAATCCGTCGAGC; XPF#2: GCTGGAGTACGAGCGACAGC. The gRNA sequences were cloned into the pX462-pSpCas9n(BB)-2A-Puro vector (a gift from Dr Feng Zhang) according to a standard protocol. The resulting gRNA/Cas9 expression plasmids were transfected into HeLa cells. After 24 h of transfection, cells were selected with puromycin (2 μg ml^−1^, Sigma) and the resulting single colonies were picked and expanded. The XPF knockout clones were confirmed by immunoblotting with anti-XPF antibody.

### TAP of SFB-tagged protein complexes and mass spectrometry

HEK293T cells stably expressing SFB-tagged TIP60 or XPF were lysed in NETN buffer (20 mM Tris-HCl [pH 8.0], 100 mM NaCl, 1 mM EDTA, and 0.5% Nonidet P-40) containing protease inhibitors on ice for 30 min. Crude lysates were clarified by centrifugation at 15,000 × *g*, 4 °C for 10 min and the resulting supernatant was incubated with streptavidin-conjugated beads (GE Healthcare) at 4 °C for 3 h on the rotator. After three times washing with NETN buffer, the bead-bound proteins were eluted with biotin (2 mg ml^−1^, Sigma). The elutes were then incubated with S-protein beads (EMD Millipore) at 4 °C for 3 h on the rotator. After three times washing with NETN buffer, the immunocomplexes were boiled in 2 X SDS loading buffer, resolved on SDS-PAGE, and analyzed by mass spectrometry. TAP of TIP60 or XPF was performed once.

Digested peptides were dissolved in buffer A (1.9% acetonitrile, 0.1% formic acid, 98% H_2_O) and loaded on analytical column (150 μm × 15 cm, 1.9 μm C18) with Easy-nLCsystem. Samples were analyzed with a 60 min gradient at flow rate 300 nl min^−1^ as follows: 8–11% buffer B (99.9% acetonitrile, 0.1% formic acid) for 8 min, 11–20% buffer B for 40 min, 20–30% buffer B for 20 min, 30–95% buffer B for 1 min, 95% B for 6 min. Thermo Orbitrap Fusion mass spectrometer was operated in data-dependent mode with one full MS scan at *R* = 120,000 (m/z 200), followed by twenty HCD MS/MS scans at *R* = 15,000, NCE = 27, with an isolation width of 1.6 m/z. The AGC targets for MS1 and MS2 scans were 3 × 10^6^ and 2 × 10^4^, respectively, and the maximum injection time for MS1 and MS2 were 80 ms and 20 ms, respectively. Dynamic exclusion was set to 45 s. Mass spectrometry data were searched by MaxQuant (version 2.3). Human protein database was downloaded from NCBI (https://www.ncbi.nlm.nih.gov/). Tolerance of precursor ions and fragment ions were set to 10 ppm and 20 ppm, and maximum miscleavage sites was 2. For pull-down experiments, variable modifications were Acetyl (Protein N-term), Oxidation (M). For acetyl proteomics experiments, variable modifications were Acetyl (Protein N-term), Oxidation (M) and Acetyl (K). FDR of peptide lever and protein level were both 1%.

### Plasmids and transfection

Most of the cDNAs were cloned into pDONR201 or pDONR221 vector (Invitrogen), and subsequently were transferred to gateway-compatible destination vectors. Point or deletion mutants were generated by site-directed mutagenesis kit (Stratagene) according to the manufacturer’s instructions. SFB-tagged SIRT1 plasmid was a gift from Dr Ja-Eun Kim (Kyung Hee University, Korea). All plasmids used in this study were verified by sequencing. Plasmids were transfected into human cells using polyetherimide.

### Co-immunoprecipitation and western blotting

HEK293T cells were transiently transfected with SFB- and/or myc-tagged expression vectors. After 24 h of transfection, cells were washed once with 1 X PBS, lysed with NETN buffer (20 mM Tris-HCl, pH 8.0, 100 mM NaCl, 1 mM EDTA, 0.5% Nonidet P-40) containing 20 mM NaF, and 1 μg ml^−1^ of pepstatin A and aprotinin on ice for 30 min, and then sonicated. The resulting whole-cell lysate was clarified by centrifugation and the supernatant (~0.3 mg protein) was incubated with S-protein beads for 3 h at 4 °C on a rotator. S-protein beads were then washed three times with NTEN buffer, boiled in 2 X SDS loading buffer, and resolved on SDS-PAGE. Membranes were blocked in 5% milk in TBST buffer and then probed with indicated antibodies. All of uncropped blots are available in source data file.

### Cell survival assays

HeLa Cells (5 × 10^2^) were plated into 60-mm dishes in triplicates and incubated for 24 h before exposure to various doses of UV or MMC as indicated. After 24 h, the medium was replaced with fresh medium. Cells were then cultured for 2 weeks and colonies were fixed and stained with Coomassie blue.

### In vitro acetylation and deacetylation assays

MBP-tagged wild-type XPF or the K911R mutant proteins purified from *E. Coli* were incubated with recombinant GST-tagged TIP60 in reaction buffer (50 mM Tris-HCl pH 8.0, 50 mM NaCl, 4 mM MgCl_2_, 0.1 mM EDTA, 1 mM DTT and 10% glycerol) in the absence or presence of acetyl-CoA (2 mM) at 37 °C for 30 min. Reactions were then subjected to SDS-PAGE and immunoblotted with anti-acetyl lysine antibody. For in vitro deacetylation assay, MBP-tagged XPF purified from *E. Coli* were acetylated by recombinant GST-tagged TIP60 in vitro. Subsequently, acetylated XPF was incubated with recombinant SIRT1 in the presence of 5 mM NAD^+^ at 37 °C for 30 min. Reactions were then subjected to SDS-PAGE and immunoblotted with anti-acetyl lysine antibody.

### Local UV irradiation

HeLa cells on coverslips were covered with an Isopore polycarbonate membrane filter (pore size, 5 μm; Millipore) and were then UV irradiated (254 nm). The filter was then removed and cells were cultured for the indicated time. After washing with PBS, cells were fixed with 3% formaldehyde for 10 min, permeabilized with 0.5% Triton X-100 for 5 min, and treated with 2.5 M HCl for 10 min to denature the DNAs. Subsequently, cells were blocked with BSA and incubated with primary anti-CPD or anti-6-4PP antibodies for 30 min at room temperature. After three times washing with PBS, secondary antibody was incubated for 30 min at room temperature. Nuclear DNA were then stained with DAPI. Cell images were taken on a fluorescence microscope (Eclipse 80i; Nikon) equipped with a Plan Fluor 60 X oil objective lens (NA 0.5–1.25; Nikon) and a camera (CoolSNAP HQ2; PHOTOMETRICS) and analyzed using Adobe Photoshop CS5.

### 6-4PP- and CPD-removal assays

Cells were irradiated with UV (at 20 J m^−2^ for 6-4PP or at 10 J m^−2^ for CPD) and then allowed to repair for indicated times in fresh medium. Cells were collected and genomic DNA was purified. The remaining 6-4PP and CPD was measured by ELISA (OxiSelect^TM^ UV-induced DNA damage ELISA kit, CELL BIOLABS, INC., STA-332-C).

### Recombinant protein purification

MBP-tagged XPF, GST-tagged TIP60 or GST-tagged SIRT1 expression constructs were transformed into *E. coli* BL21. Cells were then grown at 37 °C until log phase, and were induced with 0.1 mM IPTG at 16 °C for 12 h. Subsequently, cells were harvested, resuspended in lysis buffer (20 mM Tris-HCL [PH 7.5], 300 mM NaCl, 1% Triton X-100, and 1 μg ml^−1^ each of leupeptin and aprotinin), and sonicated. The lysate was then clarified by centrifugation and the supernatant was incubated with amylose resin or glutathione-Sepharose resin for 8 h at 4 °C. After washing the beads three times with washing buffer (20 mM Tris-HCL [PH 7.5], 500 mM NaCl, 0.5% NP-40, and 1 μg ml^−1^ each of leupeptin and aprotinin), the bound proteins were used for GST pull-down assays or eluted with washing buffer containing 10 mM Maltose or 20 mM Glutathione for in vitro acetylation or deacetylation assays.

### Peptide-binding assay

XPF-K911 non-acetyl-peptides (FAEVVSKGKGKK) or acetyl-peptides (FAEVVSK (Ac)GKGKK) were conjugated to agarose beads according to the manufacturer’s instructions (Thermo Scientific, no. 20505). Beads conjugated with peptides were incubated with HEK293T whole-cell lysates at 4 °C for 4 h. Beads were then washed with NETN buffer, boiled in 2 X SDS loading buffer, and resolved on SDS-PAGE.

### Real-time PCR

Total RNA was extracted using Trizol (Ambion, Life Technologies) and reverse-transcribed into cDNA using PrimeScript RT Master Mix (Takara, no. RR036Q). Real-time PCR was conducted using a Power SYBR Green PCR Master Mix (Applied Biosystems, Thermo Fisher Scientific, no. 4367659) with ABI 7500 RealTime PCR system (Applied Biosystems). Primers used in real-time PCR were as follows: GAPDH Forward: 5′-CGAGATCCCTCCAAAATCAA-3′; GAPDH Reverse: 5′-ATCCACAGTCTTCTGGGT GG-3′; TIP60 Forward: 5′-GGGCACCATCTCCTTCT TTGA-3′; TIP60 Reverse: 5′-ACGTAGAAGAGGAAAGGGTCTG-3′; Tublin β Forward: 5′-GCTGGACCGCATCTC TGTGT-3′; Tublin β Reverse: 5′-ACCTGAGCGAACAGA GTCCA-3′; SIRT1 Forward: 5′-CCAGAACATAGACACGCTGGA-3′; SIRT1 Reverse: 5′-TCCTCGTACAGCTTCA CAGTC-3′; XPF Forward: 5′- TTGTGAGGA AACTGTATCTGTGG-3′; XPF Reverse: 5′-AGCAAGCATGGTAGGTGTCA-3′; ERCC1 Forward: 5′- GCGACGTAATTCCCGA CTATG-3′; ERCC1 Reverse: 5′- GACCCGCAAGGCGAAGTT-3′.

### Statistics and reproducibility

Data in bar and line graphs represent mean ± SEM. Information about statistical tests is provided in the figure legends. All western blot assays shown in this study were successfully repeated at least three times. Significance is indicated by asterisk (*P < 0.05, **P < 0.01, ***P < 0.001) and *P* value < 0.05 was considered statistically significant.

### Reporting summary

Further information on research design is available in the Nature Research Reporting Summary linked to this article.

## Supplementary information

Supplementary Information

Description of Additional Supplementary Files

Supplementary Data 1

Supplementary Data 2

Reporting Summary

## Data Availability

Mass spectrometry results are provided in Supplementary Data 1 and Supplementary Data 2, however the raw mass spectrometric data are no longer available. Source Data file contains the raw data underlying all reported averages in graphs and charts, and uncropped versions of blots presented in the figures. Additional information is available from the authors upon reasonable request.

## Source data

Source Data
