# Supplementary material for: Acetylation of XPF by TIP60 facilitates XPF-ERCC1 complex assembly and activation
Source: Nat Commun. 2020 Feb 7;11:786. doi: 10.1038/s41467-020-14564-x (PMC7005904; doi:10.1038/s41467-020-14564-x)
Supplement: Supplementary file 1 — Supplementary Information [file 41467_2020_14564_MOESM1_ESM.pdf]

**Supplementary Information**

# Acetylation of XPF by TIP60 facilitates XPF-ERCC1 complex assembly and activation

Jiajia Wang[1], Hanqing He[2], Binbin Chen[1], Guixing Jiang[3], Liping Cao[3], Haiping Jiang[4], Guofei Zhang[5], Jianxiang Chen[6], Jun Huang[2], Bing Yang[2], Chun Zhou[1], and Ting Liu[1,5,*]

[1]Department of Cell Biology, and Department of General Surgery of Sir Run Run Shaw Hospital, Zhejiang University School of Medicine, Hangzhou, 310058, China

[2]Life Sciences Institute and Innovation Center for Cell Signaling Network, Zhejiang University, Hangzhou, Zhejiang 310058, China

[3]Department of General Surgery, Sir Run Run Shaw Hospital, Zhejiang University School of Medicine, Hangzhou, Zhejiang 310058, China

[4]Department of Medical Oncology, The First Affiliated Hospital, Zhejiang University School of Medicine, Hangzhou, Zhejiang 310058, China

[5]Department of Thoracic Surgery, The Second Affiliated Hospital, Zhejiang University School of Medicine, Hangzhou, Zhejiang 310058, China

[6]Department of Radiotherapy, Institute of Cancer Research and Basic Medical Sciences of Chinese Academy of Sciences, Cancer Hospital of University of Chinese Academy of Sciences, Zhejiang Cancer Hospital, Hangzhou, Zhejiang 310022, China

*To whom correspondence should be addressed: liuting518@zju.edu.cn

Keywords: Acetylation; Nucleotide Excision Repair; Interstrand Crosslink Repair; XPF-ERCC1; TIP60

Running title: TIP60 regulates XPF-ERCC1 complex assembly and activation.

Supplementary Figure 1

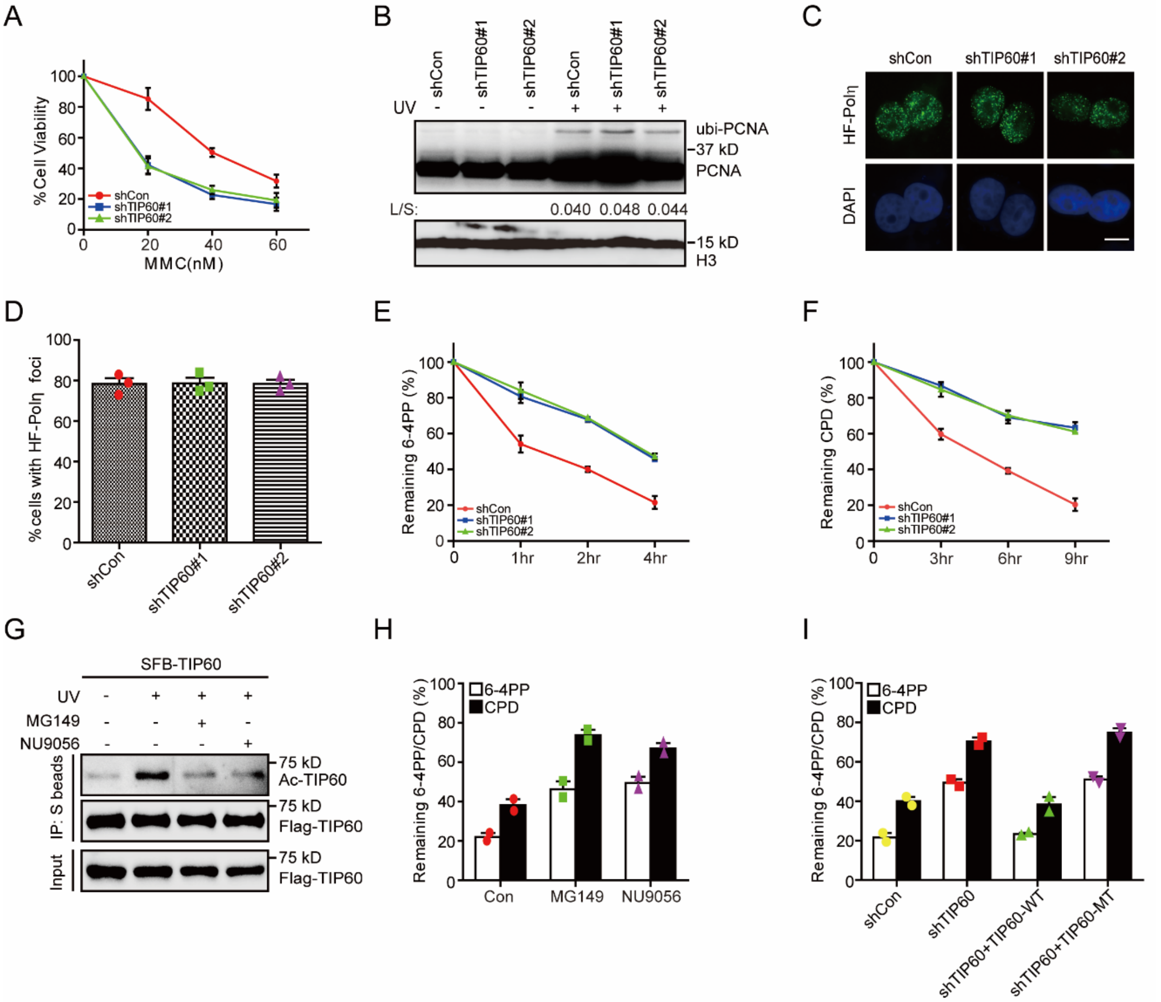


**Supplementary Figure 1. TIP60 is not required for TLS.** (**A**) The indicated cells were treated with the indicated doses of MMC. Cell survival assays were performed in triplicates. (**B**) The indicated cells were untreated or treated with 40 J m$^{-2}$ UV and allowed to recover for 4 hr. The levels of the indicated proteins were analyzed by Western blot. The ratio between long (monoubiquitinated PCNA) and short (PCNA) forms (L/S) was obtained by using ImageJ Software and shown below the blot. (**C-D**) The indicated cells were transfected with HA-Flag-Polη (HF-Polη), treated with 40 J m$^{-2}$ UV, and allowed to recover for 4 hr before being processed for Polη immunofluorescence. Representative Polη foci were shown (**C**). Data represent means ± SEM from three independent experiments (**D**). More than two hundred cells were counted in each Experiment. (**E-F**) The indicated

cells were irradiated with UV (20 J $m^{-2}$ for 6-4PP or 10 J $m^{-2}$ for CPD) and allowed to recover for indicated times. Genomic DNA was purified and subjected to ELISA to measure the remaining of 6-4PP and CPD. (**G**) HEK293T cells expressing SFB-TIP60 were treated with DMSO or treated with MG149 (100 μM) or NU9056 (20 μM) for 30 min before they were irradiated with 40 J $m^{-2}$ UV and allowed to recover for 6 hr. (**H**) HeLa cells were either treated with DMSO or treated with MG149 (100 μM) or NU9056 (20 μM) for 30 min before they were irradiated with UV (20 J $m^{-2}$ for 6-4PP or 10 J $m^{-2}$ for CPD) and allowed to recover for 4 hr and 6 hr, respectively. Genomic DNA was purified and subjected to ELISA to measure the remaining of 6-4PP and CPD. (**I**) A TIP60-depleted cell line stably expressing shRNA#2-resistant wild-type TIP60 or an acetyltransferase-defective mutant was generated. The resulting cells were irradiated with UV (20 J $m^{-2}$ for 6-4PP or 10 J $m^{-2}$ for CPD) and allowed to recover for 4 hr and 6 hr, respectively. Genomic DNA was purified and then subjected to ELISA to measure the remaining of 6-4PP and CPD. Scale bar, 10 μm. Source data are provided as a Source Data file.

Supplementary Figure 2

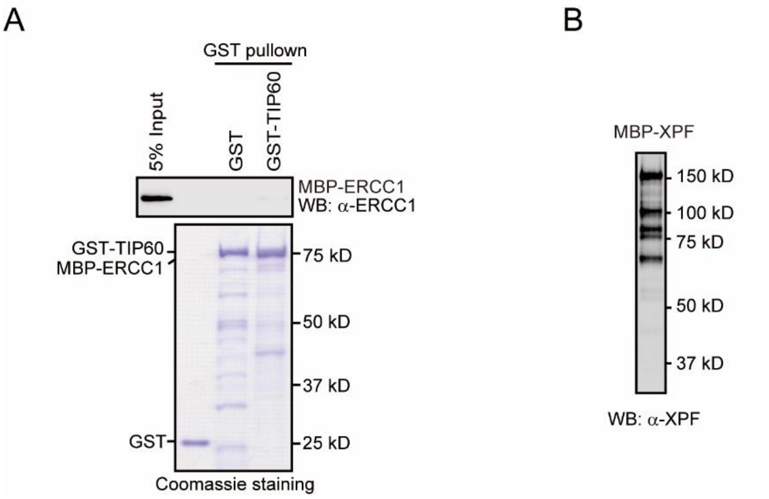


**Supplementary Figure 2.** (**A**) No direct interaction between TIP60 and ERCC1. Upper panel: ERCC1 was detected by immunoblotting. Lower panel: Proteins purified from *E. Coli* were resolved by SDS PAGE and visualized by Coomassie blue staining. (**B**) MBP-XPF purified from *E. Coli* were resolved by SDS PAGE and western blotted with anti-XPF. Source data are provided as a Source Data file.

## Supplementary Figure 3

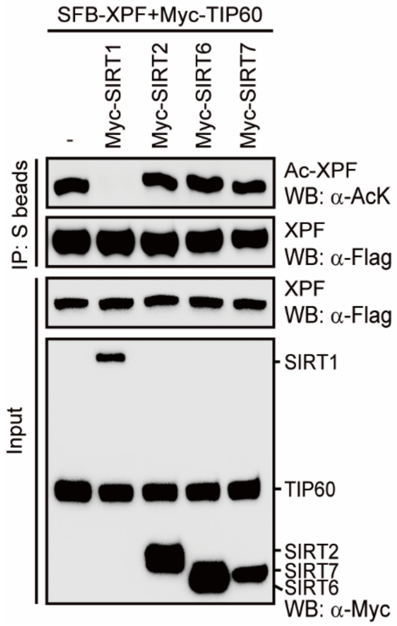


**Supplementary Figure 3.** SIRT1, but not SIRT2, SIRT6 or SIRT7, mediates XPF deacetylation. HEK293T cells were transiently transfected with the indicated plasmids for 24 hr. Whole cell lysates were then prepared and subjected to immunoprecipitation with S beads, and Western blot analysis was carried out as indicated. Source data are provided as a Source Data file.

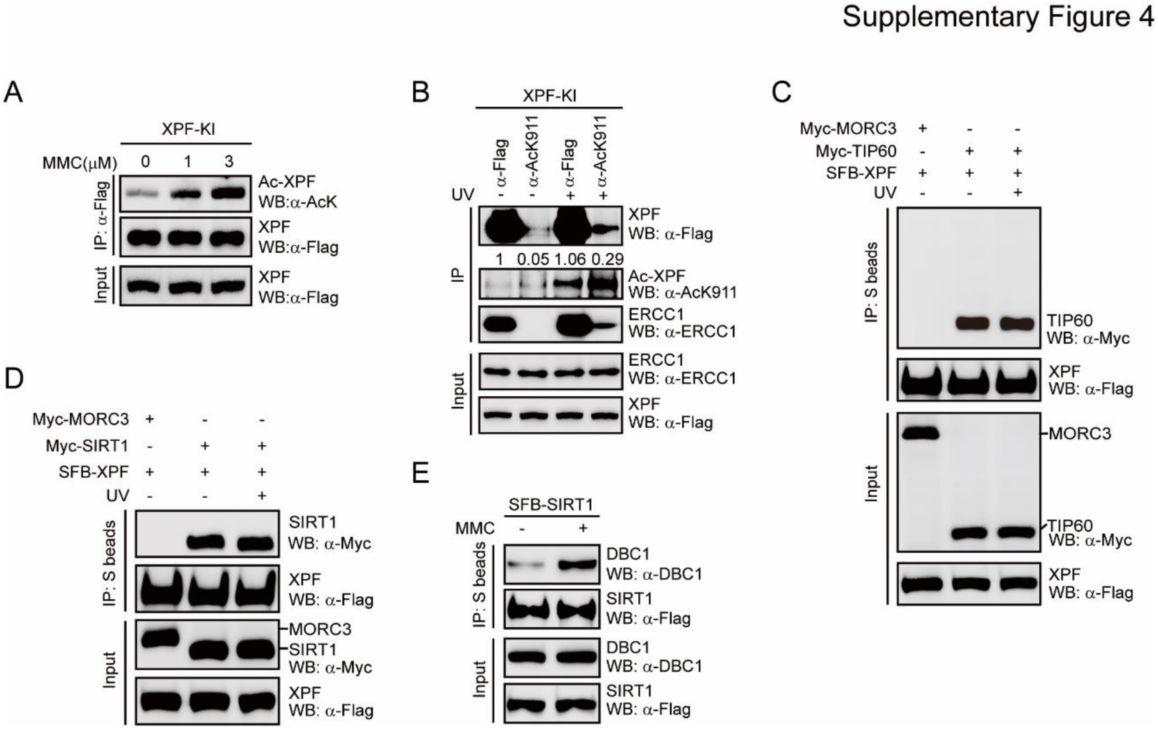


**Supplementary Figure 4.** (**A**) XPF-SFB knock-in HeLa cells were treated with indicated doses of MMC and harvested 12 hr later. Whole cell lysates were then incubated with protein A agarose beads conjugated with anti-Flag antibody, and Western blot analysis was carried out as indicated. (**B**) XPF-SFB knock-in HeLa cells were treated with 40 J $m^{-2}$ UV and allowed to recover for 4 hr. Cell lysates were adjusted on the basis of XPF and ERCC1 protein levels before being subjected to IP and then WB. Band intensity was quantified by using ImageJ Software and shown below the blots. (**C-D**) HEK293T cells transfected with the indicated plasmids were treated with 40 J $m^{-2}$ UV and allowed to recover for 2 hr. Whole cell lysates were subjected to immunoprecipitation with S beads, and Western blot analysis was carried out as indicated. (**E**) DBC1-SIRT1 interaction increases following MMC treatment. HEK293T cells transfected with SFB-SIRT1 were treated with 5 μM MMC for 6 hr. Whole cell lysates were subjected to immunoprecipitation with S beads, and Western blot analysis was carried out as indicated. Source data are provided as a Source Data file.

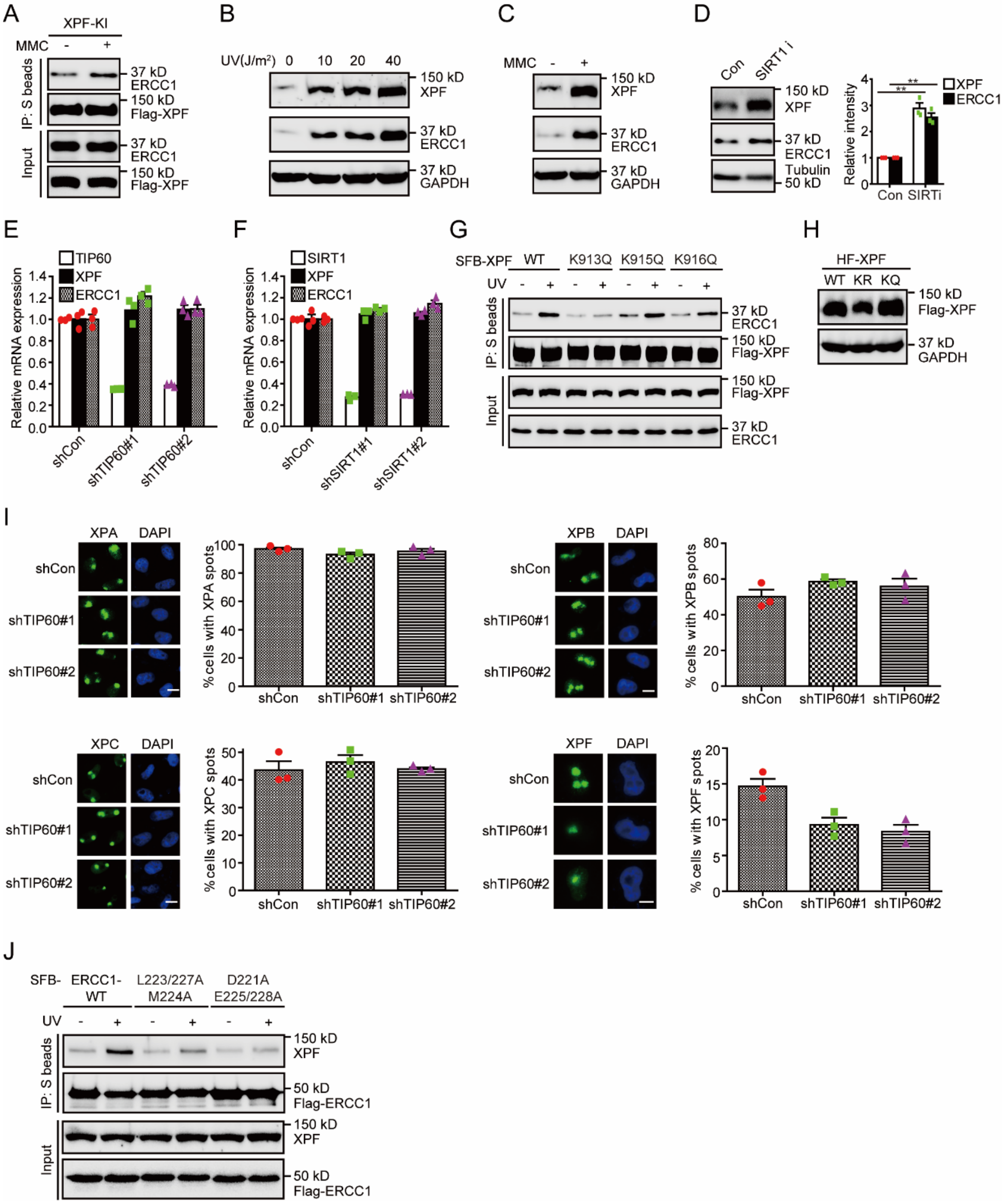


**Supplementary Figure 5. UV irradiation or MMC treatment stimulates XPF acetylation.** (**A**) XPF-SFB knock-in HeLa cells were treated with 5 μM MMC for 6 hr. Whole cell lysates were subjected to immunoprecipitation with S beads, and Western blot analysis was carried out as indicated. (**B-C**) HeLa cells were treated with UV or 5 μM

MMC and allowed to recover for 2 or 6 hr respectively. The levels of the indicated proteins were analyzed by Western blot. (**D**) HeLa cells were treated with EX527 (20 μM) for 6 hr. XPF and ERCC1 levels relative to Tubulin were quantified by ImageJ software (graph on the right). Data represent means ± SEM from three independent experiments. $^{**}P < 0.01$, Two-way ANOVA with Bonferroni post-tests. (**E-F**) HeLa cells were infected with lentiviruses carrying non-target control, TIP60- or SIRT1-specific shRNAs. The levels of specific mRNAs were determined by quantitative RT-PCR. Bars represent the average of three experiments, and error bars were presented as means ± SEM. (**G**) HEK293T cells transfected with the indicated plasmids were treated with 40 J $m^{-2}$ UV and allowed to recover for 2 hr. Whole cell lysates were subjected to immunoprecipitation with S beads, and Western blot analysis was carried out as indicated. The amounts of plasmids for transfection were adjusted to ensure comparable protein levels. (**H**) Whole cell lysates were prepared from XPF-deficient cells stably expressing HA-Flag-tagged wild-type XPF, K911R, or K911Q mutant and subjected to immunoprecipitation with anti-Flag antibody, and Western blot analysis was carried out as indicated. (**I**) HeLa cells (for XPA) or HeLa cells transfected with SFB-tagged XPB, XPC, or XPF were irradiated with UV through 5-micron filters and allowed to recover for 15 min before being stained with anti-XPA or anti-Flag antibody. Data represent means ± SEM from three independent experiments. (**J**) HEK293T cells transfected with the indicated plasmids were treated with 40 J $m^{-2}$ UV and allowed to recover for 2 hr. The amounts of plasmids for transfection were adjusted to ensure comparable protein levels. Scale bar, 10 μm. Source data are provided as a Source Data file.

Supplementary Figure 6

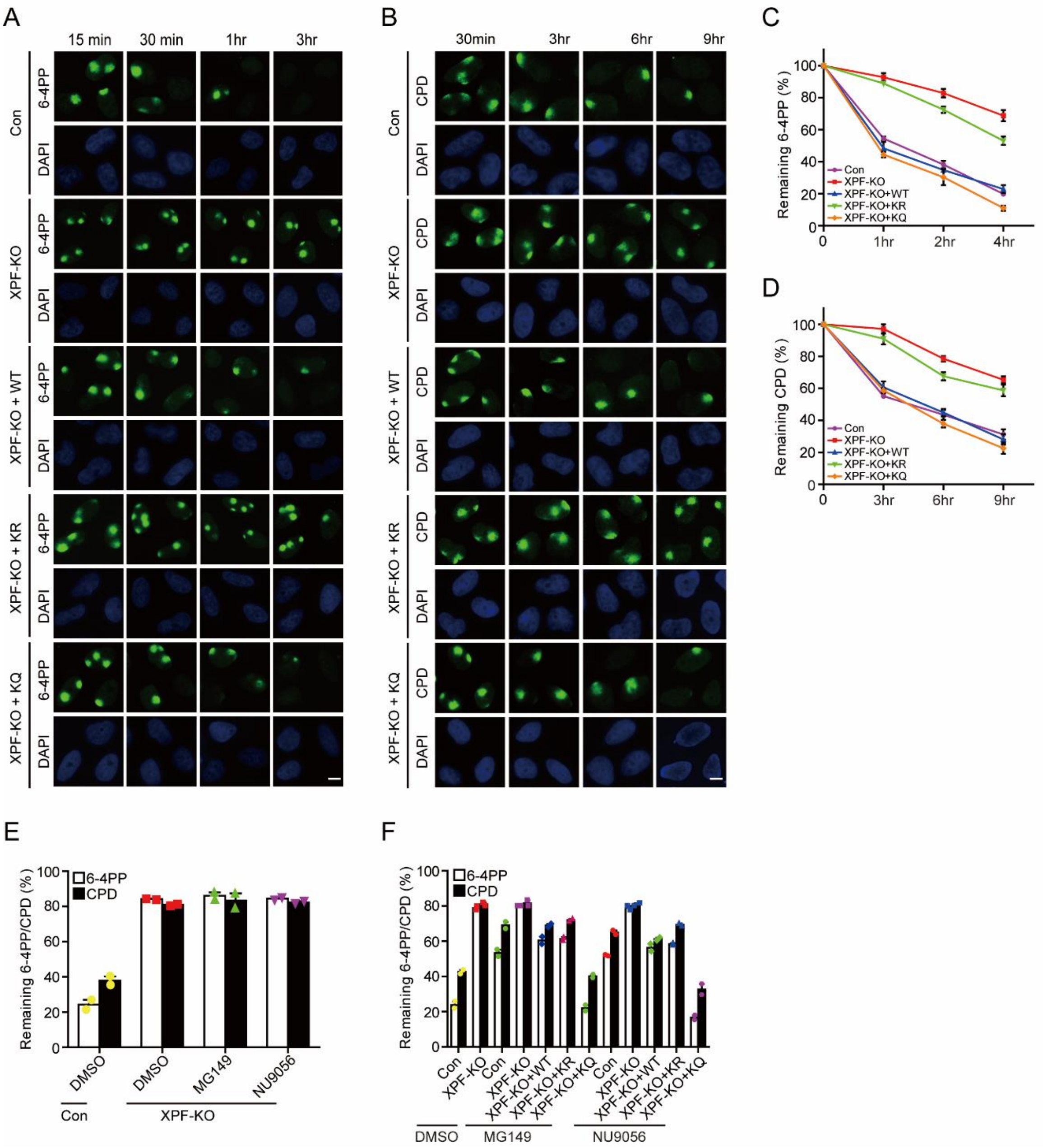


**Supplementary Figure 6. XPF acetylation promotes NER.** (**A-B**) XPF-deficient cells transfected with the indicated plasmids were irradiated with UV through 5-micron filters and allowed to recover for the indicated times before being stained with antibodies for 6-4PP (**A**) or CPD (**B**). (**C-D**) XPF-deficient cells transfected with the indicated plasmids were irradiated with UV (20 J $m^{-2}$ for 6-4PP or 10 J $m^{-2}$ for CPD) and allowed to recover for indicated times. Genomic DNA was purified and then subjected to ELISA to measure

the remaining of 6-4PP and CPD. (**E**) XPF-deficient cells were either mock treated with DMSO or treated with TIP60-specific inhibitors MG149 (100 μM) or NU9056 (20 μM) for 30 min before they were irradiated with UV (20 J $m^{-2}$ for 6-4PP or 10 J $m^{-2}$ for CPD) and allowed to recover for 4 hr and 6 hr, respectively. Genomic DNA was purified and then subjected to ELISA to measure the remaining of 6-4PP and CPD. (**F**) XPF-deficient cells transfected with the indicated plasmids were irradiated with UV (20 J $m^{-2}$ for 6-4PP or 10 J $m^{-2}$ for CPD) and allowed to recover for 4 hr and 6 hr, respectively. Genomic DNA was purified and then subjected to ELISA to measure the remaining of 6-4PP and CPD. Scale bar, 10 μm. Source data are provided as a Source Data file.
