# Supplementary material for: Acetylation of XPF by TIP60 facilitates XPF-ERCC1 complex assembly and activation
Source: Nat Commun. 2020 Feb 7;11:786. doi: 10.1038/s41467-020-14564-x (PMC7005904; doi:10.1038/s41467-020-14564-x)
Supplement: Supplementary file 2 — Description of Additional Supplementary Files [file 41467_2020_14564_MOESM2_ESM.pdf]

Description of Additional Supplementary Files

File Name: Supplementary Data 1
Description: Mass spectrometry analysis of TIP60 TAP products.

File Name: Supplementary Data 2
Description: Mass spectrometry analysis of XPF TAP products.
