# Supplementary material for: Acetylation of XPF by TIP60 facilitates XPF-ERCC1 complex assembly and activation
Source: Nat Commun. 2020 Feb 7;11:786. doi: 10.1038/s41467-020-14564-x (PMC7005904; doi:10.1038/s41467-020-14564-x)
Supplement: Supplementary file 5 — Reporting Summary [file 41467_2020_14564_MOESM5_ESM.pdf]

nature research

Corresponding author(s): Ting Liu

Last updated by author(s): Jan 2, 2020

Reporting Summary

Nature Research wishes to improve the reproducibility of the work that we publish. This form provides structure for consistency and transparency in reporting. For further information on Nature Research policies, see Authors & Referees and the Editorial Policy Checklist.

Statistics

For all statistical analyses, confirm that the following items are present in the figure legend, table legend, main text, or Methods section.

n/a	Confirmed	
☐	☒	The exact sample size (*n*) for each experimental group/condition, given as a discrete number and unit of measurement
☐	☒	A statement on whether measurements were taken from distinct samples or whether the same sample was measured repeatedly
☐	☒	The statistical test(s) used AND whether they are one- or two-sided<br>*Only common tests should be described solely by name; describe more complex techniques in the Methods section.*
☒	☐	A description of all covariates tested
☐	☒	A description of any assumptions or corrections, such as tests of normality and adjustment for multiple comparisons
☐	☒	A full description of the statistical parameters including central tendency (e.g. means) or other basic estimates (e.g. regression coefficient) AND variation (e.g. standard deviation) or associated estimates of uncertainty (e.g. confidence intervals)
☐	☒	For null hypothesis testing, the test statistic (e.g. *F*, *t*, *r*) with confidence intervals, effect sizes, degrees of freedom and *P* value noted<br>*Give P values as exact values whenever suitable.*
☒	☐	For Bayesian analysis, information on the choice of priors and Markov chain Monte Carlo settings
☒	☐	For hierarchical and complex designs, identification of the appropriate level for tests and full reporting of outcomes
☒	☐	Estimates of effect sizes (e.g. Cohen's *d*, Pearson's *r*), indicating how they were calculated

Our web collection on statistics for biologists contains articles on many of the points above.

Software and code

Policy information about availability of computer code

Data collection	Cell images were taken on a fluorescence microscope (Eclipse 80i; Nikon) equipped with a Plan Fluor 60 X oil objective lens (NA 0.5-1.25; Nikon) and a camera (CoolSNAP HQ2; PHOTOMETRICS) .
Data analysis	Cell images were analyzed using Adobe Photoshop CS5.<br>Protein levels were quantified with ImageJ software.

For manuscripts utilizing custom algorithms or software that are central to the research but not yet described in published literature, software must be made available to editors/reviewers. We strongly encourage code deposition in a community repository (e.g. GitHub). See the Nature Research guidelines for submitting code & software for further information.

Data

Policy information about availability of data

All manuscripts must include a data availability statement. This statement should provide the following information, where applicable:

- Accession codes, unique identifiers, or web links for publicly available datasets
- A list of figures that have associated raw data
- A description of any restrictions on data availability

All other data are available upon request.

Field-specific reporting

Please select the one below that is the best fit for your research. If you are not sure, read the appropriate sections before making your selection.

☒ Life sciences ☐ Behavioural & social sciences ☐ Ecological, evolutionary & environmental sciences

October 2018

For a reference copy of the document with all sections, see nature.com/documents/nr-reporting-summary-flat.pdf

Life sciences study design

All studies must disclose on these points even when the disclosure is negative.

Sample size	All experiments were repeated at least three times.
Data exclusions	No data was excluded.
Replication	All attempts at replication of the results in this study were successful.
Randomization	Samples for assay were randomly allocated.
Blinding	Blinding was used for all data acquisition and analysis.

Reporting for specific materials, systems and methods

We require information from authors about some types of materials, experimental systems and methods used in many studies. Here, indicate whether each material, system or method listed is relevant to your study. If you are not sure if a list item applies to your research, read the appropriate section before selecting a response.

Materials & experimental systems

n/a	Involved in the study
☐	☒ Antibodies
☐	☒ Eukaryotic cell lines
☒	☐ Palaeontology
☒	☐ Animals and other organisms
☒	☐ Human research participants
☒	☐ Clinical data

Methods

n/a	Involved in the study
☒	☐ ChIP-seq
☒	☐ Flow cytometry
☒	☐ MRI-based neuroimaging

Antibodies

Antibodies used	Anti-XPF polyclonal antibody was generated by immunizing rabbits with MBP-XPF (residues 616-916) fusion proteins purified from E. Coli (Hangzhou HuaAn Biotechnology Co., Ltd). Antisera were affinity-purified using AminoLink plus Immobilization and purification kit (Pierce). Anti-CPD and anti-(6-4)PP antibodies were purchased from MBL International Corporation. Anti-acetyl lysine antibody was purchased from Cell Signaling Technology. Anti-phospho-ATM (S1981), anti-ERCC1, anti-ATRIP, and anti-SIRT1 antibodies was purchased from Abcam. Anti-Myc (9E10) and anti-Flag (M2) antibodies were purchased from Covance and Sigma, respectively. Anti-GAPDH and anti-H3 antibody was purchased from Millipore. Anti-DBC1 and anti-PCNA antibodies were purchased from Bethyl Laboratories and Santa Cruz, respectively. Anti-TIP60 antibody was a gift from Dr. Yingli Sun (Beijing Institute of Genomics, China). The site-specific AcK911-XPF antibody was generated using the following peptide: aa 905-916 FAEVVSK (Ac)GKGKK.
Validation	All anti-bodies are validated by the producers for WB, IP or IF application.

Eukaryotic cell lines

Policy information about cell lines

Cell line source(s)	HeLa and HEK293T cells were from ATCC.
Authentication	N/A
Mycoplasma contamination	All cell lines used in this study have been cultured in the presence of anti-mycoplasma antibiotics (BioMycoX Mycoplasma Elimination kit, BioInd) to prevent mycoplasma contamination.
Commonly misidentified lines (See ICLAC register)	Not present in the study.
